# Morphological, physiological, and transcriptional responses of the freshwater diatom *Fragilaria crotonensis* to elevated pH conditions

**DOI:** 10.3389/fmicb.2022.1044464

**Published:** 2022-11-25

**Authors:** Brittany N. Zepernick, David J. Niknejad, Gwendolyn F. Stark, Alexander R. Truchon, Robbie M. Martin, Karen L. Rossignol, Hans W. Paerl, Steven W. Wilhelm

**Affiliations:** ^1^Department of Microbiology, University of Tennessee, Knoxville, TN, United States; ^2^Institute of Marine Sciences, University of North Carolina at Chapel Hill, Morehead City, NC, United States

**Keywords:** harmful algal blooms, climate change, lake basification, pH stress, diatoms

## Abstract

Harmful algal blooms (HABs) caused by the toxin-producing cyanobacteria *Microcystis* spp., can increase water column pH. While the effect(s) of these basified conditions on the bloom formers are a high research priority, how these pH shifts affect other biota remains understudied. Recently, it was shown these high pH levels decrease growth and Si deposition rates in the freshwater diatom *Fragilaria crotonensis* and natural Lake Erie (Canada-US) diatom populations. However, the physiological mechanisms and transcriptional responses of diatoms associated with these observations remain to be documented. Here, we examined *F. crotonensis* with a set of morphological, physiological, and transcriptomic tools to identify cellular responses to high pH. We suggest 2 potential mechanisms that may contribute to morphological and physiological pH effects observed in *F. crotonensis*. Moreover, we identified a significant upregulation of mobile genetic elements in the *F. crotonensis* genome which appear to be an extreme transcriptional response to this abiotic stress to enhance cellular evolution rates–a process we have termed “*genomic roulette.*” We discuss the ecological and biogeochemical effects high pH conditions impose on fresh waters and suggest a means by which freshwater diatoms such as *F. crotonensis* may evade high pH stress to survive in a “basified” future.

## Introduction

Algal blooms are a symptom of an imbalanced ecosystem (Heisler et al., 2008; Watson et al., 2015), where both the biotic and abiotic characteristics of a lake’s water column are altered (Anderson, 2009; Gobler and Sunda, 2012). In the case of freshwater *Microcystis* spp.-dominated harmful algal blooms (HABs), nutrient drawdown, oxygen depletion, and increased light attenuation are well-documented consequences (Paerl et al., 2001; Verspagen et al., 2004; Lehman et al., 2013; Zepernick et al., 2022c). The effects of *Microcystis* blooms on water column pH serve as a recent addition to this growing list of consequences (Van Dam et al., 2018; Krausfeldt et al., 2019; Turner et al., 2021; Zepernick et al., 2021), raising the question of how elevated pH levels associated with blooms influence other biota. Research has demonstrated *Microcystis* spp. blooms can increase lake pH to well above 9.0 *via* the photosynthetic depletion of CO_2_ (Verspagen et al., 2014; Ji et al., 2020), a phenomenon recently termed “lake basification” (Zagarese et al., 2021; Zepernick et al., 2021). Basification events have been recorded in fresh waters including Lake Taihu, China and Lake Erie, U.S./Canada (Su et al., 2015; Wilhelm et al., 2020). In Lake Erie, the mean daily water column pH remained ≥ 9.2 for ∼30 days during a record-breaking 2015 *Microcystis* spp. bloom (Zepernick et al., 2021). Further, these pH spikes oscillated on a diel cycle, with the highest pH levels (as much as 0.5 units above ambient) coinciding with peak photosynthetic periods in the late afternoon (Krausfeldt et al., 2019). While an increase in pH may benefit *Microcystis* spp. (Sandrini et al., 2016; Krausfeldt et al., 2019) and function as a positive feedback loop for late-stage bloom maintenance (Tang et al., 2018), it serves as a potential detriment to other organisms.

A recent study suggested elevated pH conditions have the potential to negatively affect algal communities beyond pH-induced carbon limitation (Zagarese et al., 2021). In a previous investigation, we demonstrated that the freshwater diatom *Fragilaria crotonensis*, which historically bloomed during the summer in Lake Erie (Hartig, 1987), exhibited lower growth rates and silica (Si) deposition rates at pH 9.2 in both monoculture and co-culture with *M. aeruginosa* (Zepernick et al., 2021). That study identified factors which likely contributed to observed declines of diatom populations during HAB events, yet there remains a need for a comprehensive assessment of how these high pH conditions influence freshwater diatom morphology and physiology beyond growth and Si deposition rates.

In the present study, we used transcriptomics to generate hypotheses concerning the physiological response and mechanistic changes behind high-pH-induced effects in this freshwater diatom. We then performed morphological and physiological measurements to validate observed transcriptomic responses and quantitatively assess the effects of elevated pH conditions on *F. crotonensis.*

## Materials and methods

### Culture conditions

To investigate the morphological and physiological effects high pH may impose on freshwater diatoms, non-axenic monocultures of *F. crotonensis* SAG 28.96 were acclimated for 6 days to either their optimal growth pH of 7.7 (Hervé et al., 2012) or the simulated Lake Erie *Microcystis* bloom-induced basification pH of 9.2 (Krausfeldt et al., 2019), as described previously (Zepernick et al., 2021). After a 6 day acclimation, samples were filter-concentrated and inoculated (T initial − T_*i*_) into their respective pH treatments for the pH assay. All pH assays were inoculated at ∼1,500 filaments mL^–1^ in this study except for the photopigment assay, as this method required higher biomass. These samples were incubated for an additional 2 days at conditions consistent with the Lake Erie summer water column (26°C; light intensity ∼55–60 μmol photons m^–2^ s^–1^ on a 12:12 h light dark cycle) prior to sample collection on day 8 of pH exposure (T final − T_*f*_). In this study, the treatment pH of 9.2 will be referred to as “high pH” while the control treatment pH of 7.7 will be referred to as “low pH.”

### pH effects on *Fragilaria crotonensis* transcription

To assess how transcriptional activity was affected at high pH and generate preliminary hypotheses based on transcriptional findings, *F. crotonensis* cultures were inoculated at ∼1,500 filaments mL^–1^ into the respective pH treatments (*n* = 3). At T_*f*_, each replicate was collected on a 2.0-μm nominal pore-size 47-mm diameter polycarbonate filter to concentrate diatom biomass. Samples were flash frozen in liquid nitrogen and stored at −80°C until extraction. RNA extractions were performed using acid phenol-chloroform methods with ethanol precipitation (Martin and Wilhelm, 2020). Residual DNA in samples was digested using a modified version of the Turbo DNase protocol and the Turbo DNA-free kit (Ambion, Austin, TX, USA). Removal of genomic DNA was confirmed *via* the absence of an amplicon band in an agarose gel after 30 cycles of PCR amplification using 519F/785R 16s rRNA primers as reported previously (Zepernick et al., 2022a). Final RNA concentrations were determined using the HS Qubit RNA assay (Invitrogen, Wltham, MA, USA). Sample library prep (Poly-A selection) and Illumina NovaSeq 6000 platform sequencing (∼25 million reads, 100 bp, paired end) were performed at Hudson Alpha (Discovery Life Sciences, Huntsville, AL, USA). Sequencing data were interleaved, filtered, and trimmed using CLC Genomics Workbench default settings (v.20) (Qiagen Digital Insights, Redwood City, CA, USA). The quality of trimmed reads was confirmed using FastQC (v.0.11.9) (Babraham Institute, Cambridge, UK). BBMap.sh (default settings) was used to remove residual rRNA reads and BBMap.repair (default settings) was used to validate paired-end reads (Bushnell, 2014). Sorted reads were mapped to the annotated *F. crotonensis* reference genome (Zepernick et al., 2022b) in CLC (default settings: length fraction: 0.5, similarity fraction 0.8) (Supplementary Data Sheet 1a), and normalized to transcripts per million (TPM). To calculate similarity (Bray–Curtis) and identify contributors to gene-expression differences between samples, non-metric multi-dimensional scaling (nMDS) and Similarity Percentage (SIMPER) analyses were performed on normalized expression values (TPM) using PRIMER (v.7) (Clarke and Gorley, 2015). Differential Expression (DE) analyses were performed in CLC, and the results were stringently filtered by significance (FDR-corrected *p*-value ≤ 0.05, log_2_ | fold-change| > 2), with predicted genes of hypothetical or unknown functions omitted from downstream analyses. Heat maps were constructed *via* heatmapper.ca (Clustering method: Average linkage, Distance measurement method: Pearson) (Babicki et al., 2016) using standardized expression scores in which genes were grouped using gene descriptions based on EggNOG and COG categories pre-assigned by the EggNOG annotation database (Huerta-Cepas et al., 2019). Manual categorization of genes was further performed based on KEGG Mapper and the KEGG Orthology (KO) database (Kanehisa et al., 2016).

### pH effects on *Fragilaria crotonensis* morphology

To directly test the morphological effects of high pH hinted at within our transcriptomes, *F. crotonensis* cultures were inoculated at ∼1,500 filaments mL^–1^ into their respective pH treatments (*n* = 5). At T_*f*,_ filament morphology was assessed with a FlowCAM 8000 imaging system using the 10x objective with a particle per used image < 1.7 (PPUI) (FlowCAM 8000, Yokogawa Fluid Imaging Technologies, Scarborough, ME, USA). Briefly, ∼1,000 *F. crotonensis* filaments per biological replicate were individually analyzed using the automated functions of the instrument with the following parameters measured: area (μm^2^), biovolume (μm^3^), length (μm), width (μm), roughness, and average green content.

### pH effects on *Fragilaria crotonensis* photopigment composition

To test for photopigment changes hypothesized from the transcriptome, *F. crotonensis* cultures were inoculated at ∼20,000 filaments mL^–1^ into their respective pH treatments (*n* = 5). At T_*f*_, measurements were made for total filaments mL^–1^ by flow cytometry CytoFLEX S (Beckman Coulter, Brea, CA, USA), equipped with the blue laser (488 nm, 50 mW), and red laser (638 nm, 50 mW) with populations gated on PerCP and FSC-H. Chlorophyll *a* (Chl *a*) autofluorescence was measured using a Turner Designs TD-700 fluorometer, equipped with a “blue” mercury bulb, a #10-050R excitation filter (λ = 340–500 nm), and a #10–115 (λ = 680 nm) emission filter. A solid standard (Turner Designs #7000–994, Turner Designs Inc., Sunnyvale, CA, USA) was used to calibrate the fluorometer prior to each day’s measurements. Subsequently, 20 mL of each culture was collected on 47-mm diameter GF/F (Whatman) filters for pigment extraction. Samples were stored at −20°C prior to pigment extraction and high-performance liquid chromatography (HPLC) analysis at *University of North Carolina Chapel Hill-Institute of Marine Sciences* as described previously (Paerl et al., 2014). Briefly, photopigment samples were extracted in 100% acetone, sonicated, and stored at −20°C for ∼24 h. The extracts (200 μL) were next assessed *via* HPLC as described previously (Van Heukelem et al., 1994; Pinckney et al., 1996, 1998, 2001). Photopigments were identified based on absorption spectra, which were determined from commercially obtained pigment standards (DHI, Hørsholm, Denmark). Net pigment concentrations (μg L^–1^) were normalized to T_*f*_ filament counts (filaments L^–1^) and mean filament length (μm) per pH treatment.

### pH effects on *Fragilaria crotonensis* photosynthetic physiology

To further evaluate the potential effects of pH on photosynthetic physiology as derived from our transcriptomes, *F. crotonensis* cultures were inoculated at ∼1,500 filaments mL^–1^ into the respective pH treatments (*n* = 5). At T_*f*_, photosynthetic efficiency metrics were assessed *via* Pulse-Amplitude-Modulation (PAM) fluorometry with the brown algae (diatom) taxa setting (Phyto-PAM-II Compact Version, Heinz Walz GmbH, Effelitrich, Germany). Samples were dark-acclimated for 20 min prior to Phyto-PAM readings as described previously (Ritchie, 2008; Torres et al., 2014; Gleich et al., 2020). After dark-acclimation, replicates were exposed to a white saturating light pulse (5,000 μmol m^–2^ s^–1^ PAR) prior to determination of the maximum theoretical photochemical quantum yield of photosystem II (PSII) (F_*v*_ • F_*m*_^–1^). Subsequently, samples were exposed to rapid light curve measurements to determine the relative maximum rate of electron transport through PSII (rETR_*max*_, μmol electrons/m^2^ s^1^). Rapid light curves were run in fourteen steps. For each step, cultures were exposed to increasing actinic irradiances starting from 1 μmol m^–2^ s^–1^ PAR, until a maximum of 1,257 μmol m^–2^ s^–1^ PAR was reached. The steps were run in 20 s intervals and a saturating pulse of 5,000 μmol m^–2^ s^–1^ PAR was run after each step. The light intensity at which saturation of PSII occurs (I_*K*_, μmol photons m^–2^ • s^–1^) was also determined.

### Statistical analyses

**FIGURE 1:**
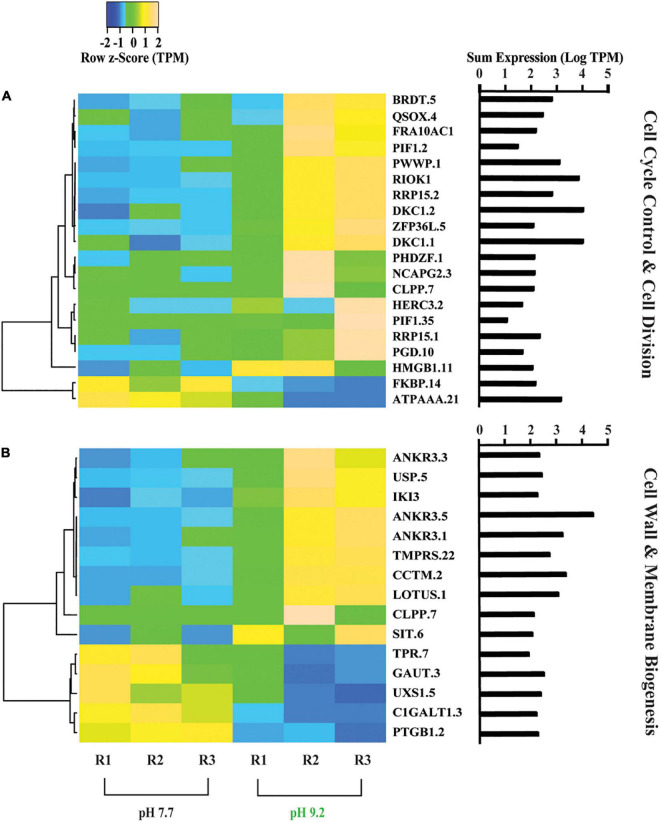
Heat maps depicting differentially expressed genes relating to *F. crotonensis* growth and cell wall morphology. **(A)** Genes in the COG category “Cell cycle control, cell division, and chromosome partitioning”. **(B)** Genes in the COG category “Cell wall, membrane, envelope biogenesis”. Cladogram clustering is to demonstrate similarity in expression. All TPM values were row z-scored, with increases in proportional transcript abundance indicated in yellow, and decreases in proportional transcript abundance indicated in blue. The sum of transcripts across all treatments (LogTPM) is indicated for each gene.

**FIGURE 2:**
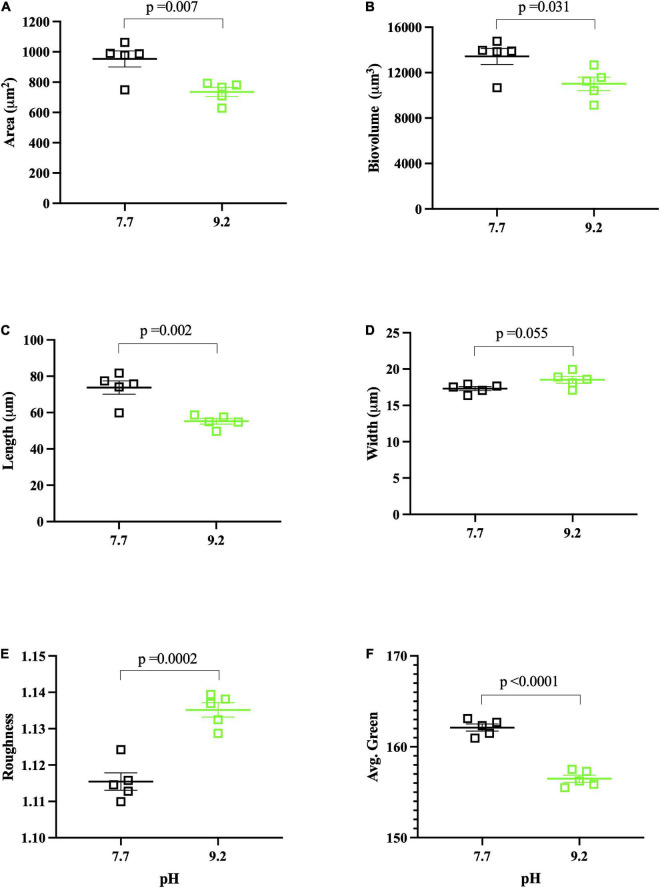
FlowCAM pH assay results collected at (T_f_). pH 7.7 replicates denoted by open, black squares. pH 9.2 replicates denoted by open, green squares. Each data point represents the mean of ∼1000 filaments per biological replicate, with the group mean of treatment replicates indicated by the central bar, and variability indicated by error bars representing the standard error of the mean (SEM). Variance for individual dots is reported in Supplementary Data Sheet 2. **(A)** Mean area (μm^2^) of *F. crotonensi*s filaments. **(B)** Mean biovolume (μm^3^) of *F. crotonensi*s filaments. **(C)** Mean length (μm) of *F. crotonensi*s filaments. **(D)** Mean width (μm) of *F. crotonensi*s filaments. **(E)** Mean roughness (dimensionless) of *F. crotonensi*s filaments. **(F)** Mean green coloration of *F. crotonensi*s filaments (dimensionless). *P*-values are for comparisons between treatments.

**FIGURE 3:**
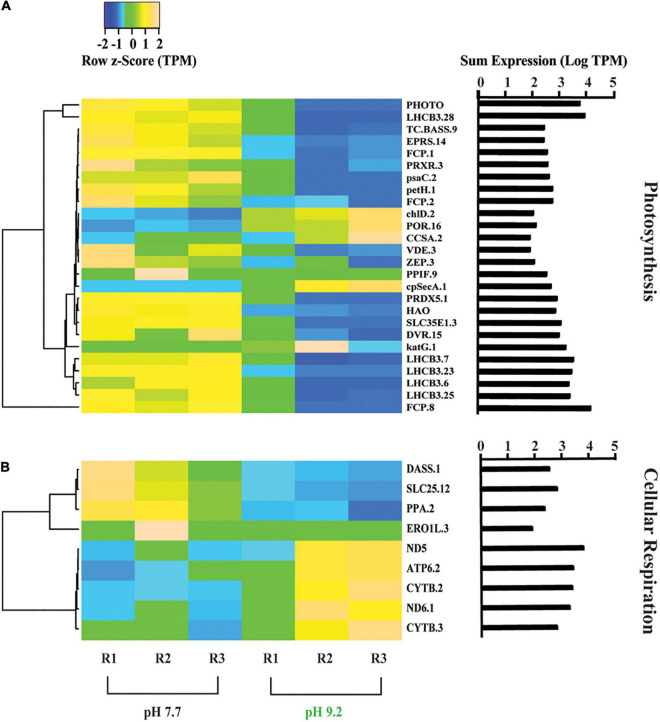
Heat map depicting differentially expressed genes relating to energy production and conversion. **(A)** Genes further sorted into photosynthesis and **(B)** cellular respiration categories. All TPM values were row z-scored, with increases in proportional transcript abundance indicated in yellow, and decreases in proportional transcript abundance indicated in blue. Cladogram clustering is to demonstrate similarity in expression. The sum of transcripts across all treatments (LogTPM) is indicated for each gene.

**FIGURE 4:**
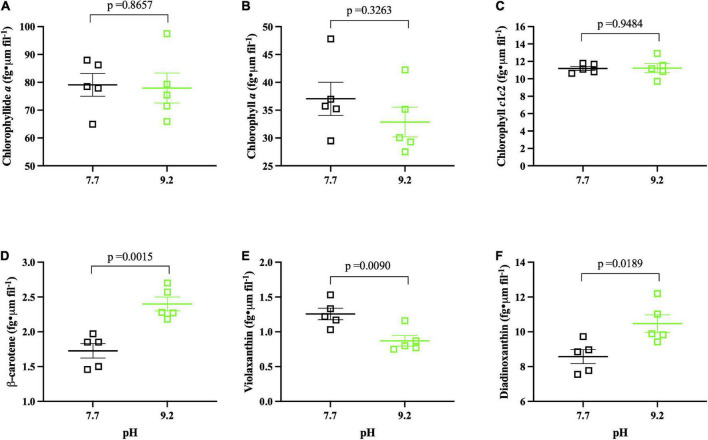
Photopigment pH assay results (*n* = 5) collected at (T_f_). pH 7.7 replicates denoted by open, black squares. pH 9.2 replicates denoted by open, green squares. The group mean of treatment replicates indicated by the central bar and variability is indicated by error bars representing the standard error of the mean (SEM). **(A)** Chlorophyllide *a* (fg • μm fil^– 1^) of *F. crotonensi*s filaments. **(B)** Chlorophyll *a* (fg • μm fil^– 1^) of *F. crotonensi*s filaments. **(C)** Chlorophyll *c*1*c*2 (fg • μm fil^– 1^) of *F. crotonensi*s filaments. **(D)** β-Carotene (fg • μm fil^– 1^) of *F. crotonensi*s filaments. **(E)** Violaxanthin (fg • μm fil^– 1^) of *F. crotonensi*s filaments. **(F)** Diadinoxanthin (fg • μm fil^– 1^) of *F. crotonensi*s filaments. *P*-values are for comparisons between treatments.

**FIGURE 5:**
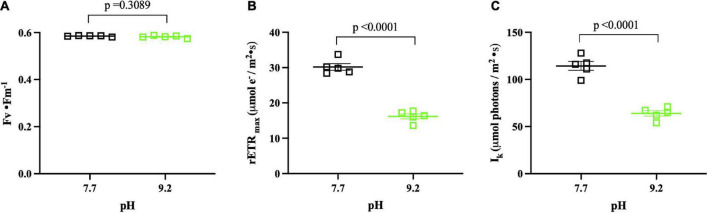
PhytoPAM pH assay results (*n* = 5) collected at (T_f_). pH 7.7 replicates denoted by open, black squares. pH 9.2 replicates denoted by open, green squares. The group mean of treatment replicates indicated by the central bar and variability is indicated by error bars representing the standard error of the mean (SEM). **(A)** Fv • Fm^– 1^ of *F. crotonensis* filaments. **(B)** rETR_max_ (μmol electrons/m^2^ • s^1^). **(C)** Photon flux at which light saturation of photosynthesis occurs I_k_ (μmol photons m^– 2^ • s^– 1^). *P*-values are for comparisons between treatments.

**FIGURE 6:**
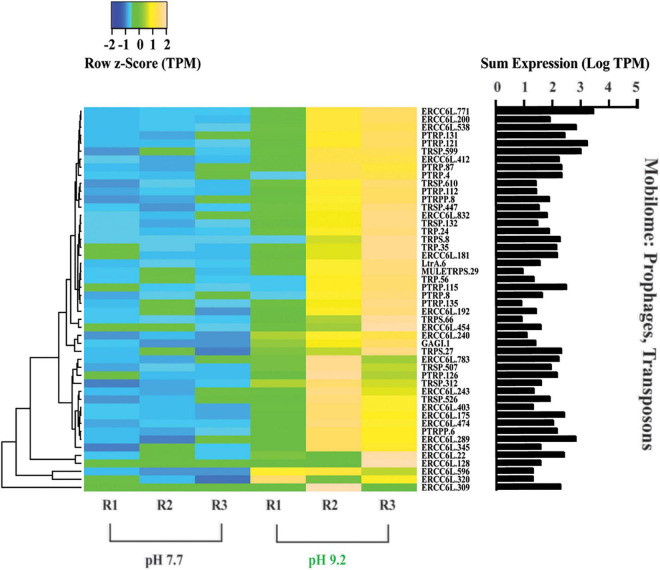
Heat map depicting differentially expressed genes relating to the “Mobilome: prophages and transposons.” All TPM values were row z-scored, with increases in proportional transcript abundance indicated in yellow, and decreases in proportional transcript abundance indicated in blue. Cladogram clustering is to demonstrate similarity in expression. The sum of transcripts across all treatments (LogTPM) is indicated for each gene.

Statistical analyses of data (Figures 2, 4, 5) were performed in GraphPad Prism (v.9.3.1) using unpaired, two-tailed *t*-tests. For this study, we consider a *p*-value < 0.05 to be significant but report all values so the reader may decide their level of risk (Supplementary Tables 1–4). Statistical comparisons of gene expression (Figures 1, 3, 6) were performed in CLC Genomics Workbench. All z-scores reported in heat maps were calculated by Heatmapper.ca. Variability in expression between replicates was assessed *via* PRIMER.

## Results

In this study, we performed transcriptomics to generate preliminary hypotheses regarding how pH may affect the physiology and morphology of *F. crotonensis*. Our transcriptomes identified 3 COG categories of genes which formed most of the differentially expressed (DE) genes between pH treatments: (1) Cell cycle control, cell division and chromosome partitioning. (2) Energy production and conversion. (3) Mobilome: transposons, prophages. Following, laboratory assays were used to test hypotheses regarding morphology and physiology for these main COG categories. Hence, our results are organized by COG category, with the transcriptome data presented first, followed by the physical laboratory assay used to test the transcriptome-derived hypotheses.

### pH induced differential gene expression between treatments

nMDS revealed an 86% similarity among low pH replicates. In contrast, high pH replicates exhibited more variability, with replicates 2 and 3 sharing 76% similarity, but replicate 1 appearing more like low pH replicates (Supplementary Figure 1). SIMPER analyses determined a mean dissimilarity of ∼27% between low and high pH replicates and identified the contribution of each gene to this variation. Notably, ∼60% of the top 50 genes driving dissimilarity were related to photosynthesis, including 17 copies of the chlorophyl a/b binding protein (LHCB3) and 8 copies of fucoxanthin-chlorophyll ac binding protein (FCP) (Supplementary Data Sheet 1b and Supplementary Figures 2A–C). Of the ∼26,000 predicted genes in the *F. crotonensis* genome, a total of 713 were differentially expressed (FDR-corrected p-value ≤ 0.05, log_2_ | fold-change| > 2) (Supplementary Figure 3A). Of these, 435 genes are annotated as either hypothetical or of unknown function (Supplementary Figure 3B). All downstream analyses focused on the 278 DE genes with predicted function (Supplementary Figure 3C and Supplementary Data Sheet 1c), of which 193 were increased in relative expression at pH 9.2 and 85 decreased in relative expression.

### High pH decreased carbohydrate transport and metabolism gene expression

Overall, genes involved in the COG category “Carbohydrate transport and metabolism” decreased in representation at high pH relative to low pH (Supplementary Figure 4). Genes involved in the Calvin-Benson-Basham (CBB) cycle decreased in expression, including glyceraldehyde 3-phosphate dehydrogenase (GAPDH), which catalyzes the single reductive step of the CBB cycle during photosynthetic carbon fixation.

Carbon metabolism genes involved in cellular respiration also decreased in relative expression at high pH. Genes involved in the Krebs cycle, such as malate/L-lactate dehydrogenase (MLDH) and divalent anion/Na^+^symporter (DASS) (Takahashi-Íñiguez et al., 2016; Lu, 2019), decreased in representation at high pH. Carbonic anhydrase (CA), which is responsible for the interconversion/acquisition of bicarbonate and CO_2_ (Sültemeyer, 1998; Burkhardt et al., 2001), decreased in expression at high pH.

### High pH increased expression of cell cycle control and cell wall biogenesis genes

Overall, the relative expression of genes categorized in “Cell cycle control, cell division, and chromosome partitioning” increased at pH 9.2 (Figure 1A). Proportional transcript abundance increased in two copies of the ribosomal RNA processing protein (RRP15) at high pH, which has been found to activate the G_1/_S checkpoint in cancer cells and thus inducing cellular arrest in G_1_ stage of interphase (Dong et al., 2017). Relative expression levels of RI0 kinase 1 (RIOK1), which is required to enter S phase (LaRonde-LeBlanc and Wlodawer, 2005), also increased at pH 9.2. Additionally, high pH increased the relative transcript abundance of butyrate response factor (ZFP36L), involved in cellular senescence and shown to induce cell cycle arrest at the G_1_ phase (Saini et al., 2020).

Genes classified within the “Cell wall, membrane, and envelope biogenesis” category also increased in overall expression (Figure 1B). A decrease in the representation of Tetratricopeptide repeat (TPR) was observed at high pH, a gene that has previously been shown to be positively correlated with silaffin expression (Frigeri et al., 2006). Further, we observed decreases in the relative expression of *N*-acetylgalactosamine 3-beta-galactosyltransferase (C1GALT1) and UDP glucuronate decarboxylase (UXS1) at high pH, both involved in glycosaminoglycan biosynthesis which has been implicated in diatom biosilicification processes (Alexander et al., 2015).

### High pH shaped *Fragilaria crotonensis* filament morphology

FlowCAM analyses showed that filaments grown at high pH had ∼20% lower mean filament area (*p* = 0.007) (Figure 2A) and mean biovolume (*p* = 0.031) (Figure 2B) than those grown at low pH. These reductions in area and biovolume were likely due to decreased filament length at high pH. Specifically, pH 9.2 *F. crotonensis* filaments had a 25% lower mean filament length (*p* = 0.002) (Figure 2C), yet mean filament width was not significantly different (*p* = 0.055) (Figure 2D). Additional FlowCAM metrics indicated pH 9.2 filaments were significantly rougher on the surface (*p* = 0.002) (Figure 2E) and significantly less green in color (*p* < 0.0001) (Figure 2F). The exact number of filaments assessed per biological replicate and the standard deviation corresponding to the mean of each biological replicate (i.e., intra-variation of each replicate) are reported in Supplementary Data Sheet 2.

### High pH decreased expression of energy production and conversion genes

Genes classified in the COG category “Energy production and conversion” were analyzed within the subcategories “Photosynthesis” and “Cellular respiration” that were created based on KEGG Mapper KO’s (Figure 3). Photosynthesis-related genes decreased in representation at high pH (Figure 3A). All 5 copies of the PSII light harvesting complex III chlorophyll a/b binding genes (LHCB) and 3 copies of the fucoxanthin-Chl binding complex genes (FCP), both involved in light absorption and energy delivery during the first step of photosynthesis (Ballottari et al., 2012), decreased in relative expression at high pH. In the chlorophyll biosynthesis pathway, magnesium chelatase subunit D (*chl*D) and NADPH:protochlorophyllide oxidoreductase (POR) increased in relative expression at pH 9.2, while divinyl chlorophyllide *a* 8-vinyl-reductase (DVR) and bifunctional glutamyl/prolyl tRNA synthetase (EPRS) decreased. In contrast, genes within the carotenoid biosynthesis pathway such as Zeaxanthin epoxidase (ZEP) and violaxanthin de-epoxidase (VDE) decreased in representation at high pH. Regarding photosynthetic metabolism, ferredoxin NADP + reductase (*pet*H) and photosystem I subunit VII (*psa*C), which are both involved in the final step of electron transfer from ferredoxin PSI to NADPH to fuel the Calvin cycle (Fischer et al., 1998; Nguyen et al., 2021), decreased in relative expression at pH 9.2. Genes involved in oxidative stress such as redoxin (PRDX5) (Hopkins and Neumann, 2019) and the catalase peroxidase gene (*kat*G.1), which quenches photosynthetically-produced ROS species (Nishiyama et al., 2001), appeared to decrease overall at high pH.

In contrast, genes classified within “cellular respiration” exhibited an overall increase in relative expression at pH 9.2 (Figure 3B). Representation of genes involved in oxidative phosphorylation increased at pH 9.2, including NADH quinone oxidoreductase chain 5 and 6 genes (ND5) (ND6) (Melo et al., 2004) and ATP synthase subunit α gene (ATP6) (Vázquez-Acevedo et al., 2016). In contrast, mitochondrial transport genes decreased in relative expression at high pH, including carrier gene (SLC25) (Ruprecht and Kunji, 2020).

### High pH modified photopigment composition

After 8 days of pH exposure, there was no significant difference in filament concentration as a function of pH treatment (*p* = 0.1745) (Supplementary Figure 5A). However, high pH replicates had significantly decreased in Chl *a* autofluorescence (*p* < 0.0001) (Supplementary Figure 5B). Normalized photopigment concentrations of Chlorophyllide *a*, Chlorophyll *a*, Chlorophyll *c*^1^*c*^2^ (fg • μm fil^–1^) (*p* ≥ 0.3263) (Figures 4A–C), and Total Chl *a* (fg • μm fil^–1^) (*p* = 0.3503) (Supplementary Figure 6) were not significantly different as a function of pH. In contrast, high pH cultures had ∼40% more β-carotene (*p* = 0.0015) (Figure 4D) and ∼20% more Diadinoxanthin (*p* = 0.0189) (Figure 4F) per cell compared to low pH replicates. Further, high pH incubated cultures demonstrated ∼30% less Violaxanthin (fg • μm fil^–1^) compared to pH 7.7 counterparts (*p* = 0.0090) (Figure 4E), yet Fucoxanthin and Neoxanthin normalized pigments did not significantly vary per cell as a function of pH (*p* ≥ 0.337) (Supplementary Figure 7).

Ratios of total carotenoids:total Chl *a* were higher at pH 9.2 but fell short of significance (*p* = 0.2207) (Supplementary Figure 8). However, β-carotene:total Chl *a* (*p* = 0.0002) and Diadinoxanthin:total Chl *a* (*p* = 0.0008) ratios were both significantly higher at pH 9.2 (Supplementary Figures 9A–C). Mean Chl *a*/Chl *c*^1^*c*^2^ ratios, which serve as a proxy for the size of the light harvesting antenna complex (Lamote et al., 2003; Nguyen-Deroche et al., 2012; Heydarizadeh et al., 2019), demonstrated a consistent downward trend at high pH, though these results were not significant (*p* = 0.4335) (Supplementary Figure 10). In summary, pH did not significantly affect chlorophyll pigment concentration per cell, but a significant effect of pH 9.2 was observed on carotenoids (β-carotene and xanthophylls) in this study.

### High pH altered *Fragilaria crotonensis* photophysiology

High pH of 9.2 did not significantly alter optimal photochemical quantum yields of photosystem II (Fv • Fm^–1^) (*p* = 0.3089) (Figure 5A). However, the relative mean maximum electron transport rate through photosystem II (rETR_*max*_) was ∼50% lower at pH 9.2 (*p* < 0.0001) (Figure 5B). Additionally, the photon flux at which light saturation of photosynthesis occurs (I_*k*_) was ∼50% lower at pH 9.2 (*p* < 0.0001) (Figure 5C).

### High pH increased expression of transposon genes

Out of the 278 DE genes in the dataset, 193 were overrepresented at the high pH treatment. Further, of these 193 DE genes which increased in relative expression at pH 9.2, 25% belong to the “Mobilome: transposons, prophages” COG category (Figure 6). All 48 genes within the Mobilome COG category increased in representation at high pH. Notably, nine copies of the plant transposon gene (PTRP) were overrepresented at high pH. No Mobilome categorized genes were decreased in relative expression at the high pH treatment within our DE dataset.

## Discussion

Harmful algal bloom-induced increases in pH have been shown in the literature for decades (Talling, 1976; Jeppesen et al., 1990; Lopez-Archilla et al., 2004; Sandrini et al., 2016), yet these studies largely focused on carbon chemistry. Few assess the consequences on other members of the biotic community. Previously, we demonstrated that growth of *F. crotonensis* decreased at pH 9.2 and discovered Si deposition declined at high pH in diatom cultures as well as natural Lake Erie communities. Here, we built on this using transcriptomics to identify physiological processes and resulting morphological changes that may be altered by high pH. Then, we employed a variety of *in vitro* lab assays to better clarify and validate our transcriptomic results and identified potential mechanisms by which these changes may be occurring. Finally, we discuss these observations within the broader ecological scope of lake basification, and potential future implications on global freshwater diatom communities.

### Evidence of carbon limitation is lacking in high pH transcriptomes

Declines in diatom viability during bloom-induced basification have historically been thought to be due to inorganic carbon-limitation. However, evidence of carbon limitation was lacking in our transcriptomes. Recent studies investigating carbon-limitation in the model diatom *Phaeodactylum tricornutum* observed increases in the expression of the CO_2_ concentrating mechanism (CCM) genes (specifically CAs) in response to low CO_2_ availability (Burkhardt et al., 2001; Heydarizadeh et al., 2019). However, there was an absence of biophysical CCM genes in our DE data set except for one CA, which was decreased at pH 9.2. Additionally, Heydarizadeh et al. (2019) concluded high light and carbon limitation increased the expression of genes associated with the biochemical CCM, yet we saw no evidence of this occurring within our transcriptomes. In addition, a prior study determined that environmental *F. crotonensis* bloom samples incubated at carbon-limited pH 9.4 did not recover photosynthetic rates after CO_2_ enrichment compared to carbon-limited controls (Talling, 1976), suggesting *F. crotonensis* may not have been exclusively carbon limited. Further, the *F. crotonensis* photosynthesis rates at pH 9.4 in both carbon-limited and carbon-enriched samples were lower when compared to general phytoplankton photosynthetic rates (Talling, 1976). In contrast, when Talling (1976) replicated the experiment with natural *Microcystis* spp. bloom samples, a significant recovery of photosynthetic rate was observed after CO_2_ enrichment. These results from Talling (1976) and others are consistent with our own; they suggest *F. crotonensis* is exhibiting decreased photosynthetic metabolism not due to pH-induced carbon-limitation alone, but due directly to alkaline pH. In summary, prior studies have induced C-limitation in diatoms and observed alterations in carbon-related genes within their transcriptomes. In our study, we did not observe these gene trends found to coincide with carbon-limitation, yet further research is needed concerning this phenomenon.

### Photostress is a likely physiological consequence of high pH in *Fragilaria crotonensis*

Previously, we demonstrated that growth and Si deposition rates decline at high pH. Yet, the mechanisms driving these observations remained unclear. The present study indicated one of the primary processes driving these effects may be photostress. Surprisingly, ∼60% of the top 50 genes contributing to pH transcriptome dissimilarity are involved in photosynthesis, with PSII and light antennae complex components forming the majority. Further, PhytoPAM data suggested a significant decrease in PSII electron flow and light saturation threshold at high pH, while pigment analysis indicated a significant increase in the photoprotective carotenoids β-carotene and diadinoxanthin. Cumulatively, this data suggests *F. crotonensis* experienced photostress at high pH.

Prior studies demonstrated that short-term photoacclimation strategies decrease photosynthetic processes *via* LHC modifications (Horton et al., 1996; Bassi and Caffarri, 2000), reductions in the size of the LHC (Perry et al., 1981), and initiation of non-photochemical quenching (NPQ) (Wilhelm et al., 2006; Bertrand, 2010). In our study, *F. crotonensis* decreased electron flow through PSII at high pH as indicated by significantly lower PSII rETR_max_ and decreased expression of genes encoding for the LHC of PSII (LHCBs and FCPS) (Ballottari et al., 2012). Additional transcriptomic data implied a corresponding decrease in PSI photosynthetic capacity at high pH, as evidenced by decreases in ferredoxin NADP + oxidoreductase (FNR) enzyme gene expression (*pet*H and *psa*C). Cumulatively, these data suggest there are alterations to photosynthetic capacity at high pH. In addition, decreases in LHCB expression have been found to serve as a photoprotective response to excessive light intensities and light saturation to prevent damage to PSII and reduce ROS generation (Thomas, 2016). In our study, this strategy appeared to be successful, as PhytoPAM data demonstrated no significant differences in (Fv • Fm^–1^) as a function of pH. Regarding LHC modifications, significant increases in diadinoxanthin photopigment composition at high pH, decreases in carotenoid biosynthesis gene expression, and increases in expression of the key regulatory gene of the chlorophyll biosynthesis pathway (*chl*D), collectively suggest *F. crotonensis* may be restructuring the pigment composition of its LHCs in response to alkaline pH. Additionally, mean Chl a/Chl *c1c2* ratios, which serve as a size proxy for the LHC, exhibited a downward trend at pH 9.2, though this was not significant. This suggested that after 8 days of alkaline pH exposure, *F. crotonensis* may have begun reducing the size of its LHC as a photoacclimation strategy. However, this would likely result in an increase in rETR_max_ as more light would then be required to drive rETR saturation, which we do not observe in this study. Hence, it is probable while *F. crotonensis* appears to be restructuring the photopigment constituents of its LHC, this does not appear to alter the LHC size overall. Moreover, we observed significant increases in diadinoxanthin at pH 9.2 and increased representative expression of thylakoid translocase subunit SecA gene (cpSecA.1), suggesting the activation of a NPQ energy dissipation pathway which is controlled by diadinoxanthin and a trans-thylakoid proton gradient (Muller et al., 2001; Bertrand, 2010). Indeed, the diadinoxanthin cycle has been described as the most important short-term photoprotective mechanism in diatoms (Bertrand, 2010). Cumulatively, these results indicate that *F. crotonensis* protects the photosynthetic membrane at high pH by employing short-term photoacclimation mechanisms such as modifying LHC composition and increasing the concentration of pigments involved in NPQ.

The photostress and photoresponse findings are supported by prior diatom studies. Park et al. (2010) determined that *Chaetoceros neogracile* alters diadinoxanthin levels and FCP expression in response to increased light intensity, and a recent study determined the diatom *P. tricornutum* alters the photopigment composition of its FCP binding complexes in response to varying light sources (Oka et al., 2020). Collectively, these prior studies suggest the high pH effects we observed mirror diatom responses to high light intensity. Yet, the light intensity was held constant throughout the entirety of our experiments, implying that growth at pH 9.2 affects *F. crotonensis’s* phototolerance. Indeed, in our study *F. crotonensis* filaments may be light saturated at pH 9.2 as evidenced by a 50% lower PSII saturation threshold (I_k_), with saturation setting in at 60 μmol photons m^–2^ s^–1^. This suggests that *F. crotonensis* is experiencing the light saturation of downstream metabolisms, with potential alterations to the light antennae serving as a secondary effect of this phenomenon. Further, photoprotective pigments increased at high pH while light harvesting pigments did not, implying filaments are prioritizing photoprotection over photon acquisition. Cumulatively, transcriptomic, morphological, and physiological evidence in our study imply *F. crotonensis* may have a lower phototolerance of light at high pH, suggesting basification pH conditions alter diatom photophysiology.

### Cell cycle arrest another physiological consequence of high pH?

High-pH-induced photostress explains altered photopigment composition and photosynthetic physiology but falls short of directly accounting for morphological changes observed in this study (i.e., differences in filament length and frustule roughness) and prior observed physiological effects (i.e., decreased growth and Si deposition rates). Our transcriptomic analyses indicate another factor may be driving these high pH effects: arrest of the cell cycle at the G_1_/S checkpoint (Jang et al., 2005). Indeed, a prior study determined this checkpoint is typically regulated by light in photosynthetic eukaryotes (Moulager et al., 2010), thus photostress may be contributing to its arrest in the diatom *F. crotonensis*. In support of this, we observed an increase in the expression of multiple genes associated with the G_1_/S checkpoint at high pH. Moreover, a recent study demonstrated cell cycle arrest in G_1_ does not lead to changes in Fv • Fm^–1^, but does result in lower rETR_max_, higher NPQ, and gene expression patterns consistent with LHC and ROS scavenging in the diatom *P. tricornutum* (Kim et al., 2017), all of which are observed in our study.

However, if photostress is inducing cell cycle arrest, how does this result in lower growth and Si deposition rates? With regard to growth, a prior diatom study indicated photosynthetic metabolism and the cell cycle are closely related, with photosynthetic capacity at its highest during the main growth phase of G_1_ (Claquin et al., 2004). Biosilicification is also linked to the cell cycle and cell growth (Hildebrand et al., 2007; Shrestha et al., 2012), with the G_1_ phase serving as the phase where diatoms reach their full size and where girdle band formation of the frustules occurs (Javaheri et al., 2014). Additional studies suggest cell growth cannot occur without girdle band formation (Crawford, 1981; Volcani, 1981; Claquin et al., 2002). Hence, we may observe decreased growth and Si deposition rates, smaller filaments, and malformed frustules due in part to this arrest and disruption in the G_1_ cell phase. It is well-established that eukaryotic phototrophs use cell cycle arrest as a means to combat stress, with this mechanism induced by a variety of abiotic stressors (Eekhout and De Veylder, 2019; Takahashi et al., 2019). We suggest the photostress observed in pH 9.2 treatments may be responsible for this apparent cell cycle arrest hinted at in our transcriptomic data, and therefore contributing to morphological and physiological effects observed in *F. crotonensis*.

### High pH results in smaller, rougher, and browner *Fragilaria crotonensis* morphologies

In this study, growth at pH 9.2 resulted in significant morphological changes within our model freshwater diatom. Filaments maintained at high pH for 8 days exhibited significantly shorter lengths, resulting in lower biovolume and area. Despite this, individual diatom cells did not significantly differ in width. A prior study demonstrated warming temperatures results in smaller diatom cells across the Laurentian Great Lakes (Bramburger et al., 2017). Considering lake basification events will likely coincide with warming temperatures, future freshwater diatom communities may exhibit both smaller filaments and smaller individual cells.

FlowCAM analyses further indicated filaments had rougher exterior surfaces at high pH. We previously examined the pH dependence of Si deposition, demonstrating that it decreased at pH 9.2. Here, we build upon this with FlowCAM and transcriptomic data which suggest diatoms are struggling to deposit Si, and the diatoms that do successfully form frustules may have malformations which are evidenced by this “roughness.” We observed a decrease in the expression of a gene (TPR) whose expression is positively correlated with silaffin expression (a gene directly involved in Si deposition) (Frigeri et al., 2006), suggesting *F. crotonensis* may be downregulating the biosilicification process (Shrestha et al., 2012). Genes suggested to encode for diatom cell wall components such as CLPP and ANKR3 (Frigeri et al., 2006), and C1GALT and UXS1 (Alexander et al., 2015), were also decreased in expression at high pH. Alterations in the expression of biosilicification genes, and/or those involved in forming the cell wall structures which the frustules sit upon, will produce morphological malformities (Round et al., 1990; Hildebrand et al., 2006). Cumulatively, these data suggest diatoms struggle to deposit Si frustules at pH 9.2, likely leading to cell walls with rougher and malformed phenotypes.

In addition to smaller and “rougher” filaments, high pH results in “browner” diatoms due to significantly increased concentrations of β-carotene and diadinoxanthin but ∼constant chlorophyll concentrations. Prior studies demonstrate both fucoxanthin (involved in light harvesting) and diadinoxanthin (involved in photoprotection) are the main carotenoids of diatoms, serving as components of the LHCs (Ballottari et al., 2012). Indeed, total carotenoid:total Chl *a* ratios were higher at pH 9.2, though these findings fell short of significance due to ∼constant fucoxanthin concentrations. Nonetheless, there were significant increases in the diadinoxanthin:total Chl *a* and β-carotene:total Chl *a* ratios observed in pH 9.2 filaments. This contradicts our transcriptomic data, as carotenoid biosynthesis genes (ZEP and VDE) were decreased in expression at pH 9.2. However, changes in pigment levels often occur without detectable changes in gene expression (Kuczynska et al., 2015). Regardless, significantly higher β-carotene and diadinoxanthin photopigment concentrations lead to “less green” filaments and significantly lower Chl *a* autofluorescence, all suggesting “browner” diatoms at pH 9.2. Cumulatively, this data demonstrates lake basification induces significant changes to *F. crotonensis* morphology, resulting in smaller, rougher, and browner filaments.

### Lake basification has ecological and biogeochemical implications

While acidification has been suggested to benefit the growth and ecological resilience of marine diatoms (Wu et al., 2014; Valenzuela et al., 2018), we suggest that basification poses a detriment. Diatoms are responsible for ∼20% of global primary production (Nelson et al., 1995), acting as an integral component of the aquatic primary producers. Hence, altering diatom populations will evoke significant biotic and biogeochemical implications within the aquatic ecosystem (Rühland et al., 2015). For example, here we demonstrate high pH results in smaller, browner, rougher diatoms. Based on prior research, we postulate that these morphological alterations will modify grazing patterns of secondary consumers and selective filtration of higher trophic consumers (Vanderploeg et al., 2001, 2013; Baranowska et al., 2013). This decrease in size will also likely increase diatom predation by Dreissenids, as smaller diatoms seem to be selectively filtered (Reavie and Barbiero, 2013). Additionally, declines in Si deposition rates combined with rougher frustules suggests thinner and malformed cell walls which may reduce their effectiveness as a defense mechanism against zooplankton grazing (Pančić et al., 2019; Ryderheim et al., 2022) and viral infection (Kranzler et al., 2019).

Due in part to their heavy Si frustules, diatoms also play an instrumental role in global biogeochemical cycles and nutrient export to the benthos (Struyf et al., 2009; Benoiston et al., 2017), with studies demonstrating the Si cycle is more strongly inter-related with the carbon, nitrogen, and phosphorus cycles than previously thought (Tréguer and De La Rocha, 2013; Tréguer et al., 2021). For example, Lake Erie undergoes such a high degree of diatom deposition to the benthos in the winter-spring, that it serves as a substantial driver of summer hypoxia (Reavie et al., 2016). Another study demonstrated that even when an estuarine water column was dominated by flagellates, the labile organic matter settling to the sediment was derived from diatoms (Haese et al., 2007). Thus, we postulate lake basification-induced changes to diatoms will have significant downstream effects within the lacustrine ecosystem and biogeochemical cycles, particularly with respect to the benthos which experiences the highest diatom-nutrient deposition.

### “*Genomic roulette*”: The key to freshwater diatom success in a basified future?

Abundant transcripts from transposable elements within the *F. crotonensis* genome provide insight into a mechanism these diatoms may use to adapt and persist at high pH or other severe environmental stressors that could be otherwise detrimental. Approximately 25% of the DE genes that were increased in expression at high pH belonged within the “Mobilome: prophages, transposons” COG classification. Mobile elements like these change the architecture of an organism’s genome by rearranging themselves to new locations or (in some cases) moving pieces of the genome itself. Such rearrangements can, for example, insert into a gene and disrupt it, or insert into a regulatory site and change how genes are expressed (Pennisi, 1998; Lisch, 2013; Schrader and Schmitz, 2019). Extensive genomic variation has been attributed to similar rearrangements in green alga like *Chlamydomonas reinhardtii* (Flowers et al., 2015).

It appears that a potential response to environmental conditions stressful enough to heavily damage an organism—like a shift to extreme pH conditions—is an attempt to make a significant, microevolutionary leap by re-arranging one’s own genomic architecture at random. Diatoms have been described as “one of the most rapidly evolving eukaryotic taxa on Earth” (Oliver et al., 2007; Vardi et al., 2009). This speed has been attributed to their high proportion of retrotransposons, long terminal repeats, and transposable elements (Bowler et al., 2008; Vardi et al., 2009; Rastogi et al., 2018). This type of win or lose strategy, which we have termed “genomic roulette,” is perhaps an extreme interpretation of the role of “jumping genes” in organismal adaptation to ecological stress (McClintock, 1956; Capy et al., 2000; Horváth et al., 2017). We define genomic roulette as an upregulation of mobile elements in response to an environmental stressor to rapidly increase mutations within an organism, and thus the genetic diversity within a population, over a short period of time. While most random mutations of this nature can be hypothesized as disadvantageous, there remains the likelihood a small subset will confer fitness to the organism and facilitate survival.

Stress-induced alterations to genomic architecture have been suggested in other phototrophs ranging from single-celled cyanobacteria (Lin et al., 2010; Hu et al., 2018) to multicellular plants (Negi et al., 2016; Roquis et al., 2021). For example, the cyanobacterium *Microcystis aeruginosa* in lab cultures has been shown to upregulate transposases when shifted to urea as a nitrogen source for growth (Steffen et al., 2014). With regard to diatoms, recent studies have suggested genome evolution was responsible for cold-climate adaptations in the polar marine diatom *Fragilariopsis cylindrus* (Mock et al., 2017) and warmer-climate adaptations in tropical marine diatoms in response to ocean warming (Jin and Agustí, 2018). Our observations provide a potential mechanism for how our freshwater diatom *F. crotonensis* may be gambling in the game of genomic roulette at high pH, with the potential to facilitate a rapid adaptation to these high pH conditions at the likely cost of many in the cohort. Going forward, there is a need to better elucidate this hypothesis within diatoms, determine if this strategy of genomic roulette extrapolates to other organisms beyond photoautotrophs, and a necessity to develop an understanding of its rate of success.

## Conclusion

In this study, transcriptomic analyses revealed that genes associated with photosynthesis, not carbohydrate metabolism, were driving dissimilarity between high pH vs. low pH expression. We demonstrated that a pH of 9.2, which is routinely reached during lake basification events, significantly alters *F. crotonensis* filament morphology resulting in smaller, browner, and rougher diatoms after just 8 days of exposure. This pH further modifies F. *crotonensis* photophysiology by significantly decreasing both relative maximum electron transport rates through PSII and light saturation thresholds. Cumulatively, these transcriptomic, morphological, and physiological findings imply *F. crotonensis* experienced photostress in the high pH treatment, with evidence suggesting filaments invoke photoacclimative strategies in response. In turn, transcriptomic evidence suggests this photostress is inducing cell cycle arrest at the G_1_/S checkpoint, which would explain the decreased growth and Si deposition rates observed previously (Zepernick et al., 2021).

Increased sensitivity to light stress is likely exacerbated in the environment, as prior findings indicate the highest diel pH spikes coincide with peak photosynthesis rates and light levels during the afternoon (Krausfeldt et al., 2019), a phenomenon likely intensified by prolonged basification events of up to ∼30 days in the environment (Zepernick et al., 2021). Yet, this stress may be partially alleviated by the thick *Microcystis* spp. scum associated with bloom events, as colonies regulate their own buoyancy and shade the water column. Thus, further research is needed to understand responses of freshwater diatoms to both diel variations in pH on a short-term scale, and the long-term effects basification will impose on freshwater diatom communities. As climate change serves to increase cyanobacterial bloom distribution, duration and frequency (Wells et al., 2020), there is a need to elucidate how freshwater diatom communities will respond (and adapt) to these lake basification events.

## Data availability statement

The *F. crotonensis* annotated nuclear genome was deposited in GenBank under the accession number: JAKSYS000000000. Data are available under BioProject accession number: PRJNA807324 and BioSample accession number: SAMN25978007. Further information concerning the genome may be accessed in Zepernick et al. (2022b). The transcriptome raw reads that were mapped to the *F. crotonensis* genome were deposited in the NCBI sequence read archive under BioProject accession number: PRJNA866361. Forward and reverse raw libraries for each treatment replicate can be accessed by the following SRA accession numbers: pH 7.7 replicate 1 forward/reverse reads SRR20853615, pH 7.7 replicate 2 forward/reverse reads SRR20853613, pH 7.7 replicate 3 forward/reverse reads SRR20853612, pH 9.2 replicate 1 forward/reverse reads SRR20853611, pH 9.2 replicate 2 forward/reverse reads SRR20853610, and pH 9.2 replicate 3 forward/reverse reads SRR20853614.

## Author contributions

BZ and DN performed all culture work, pH assays, sample collection, and FlowCAM analyses. GS performed PhytoPAM measurements. KR and HP performed photopigment extraction and HPLC analysis. BZ performed RNA extractions, QC, PRIMER analyses, statistical analyses, and heat map configuration. BZ, AT, and RM performed transcriptome analyses. All authors contributed to the drafting and final version of the manuscript.
