# Supplementary material for: Morphological, physiological, and transcriptional responses of the freshwater diatom Fragilaria crotonensis to elevated pH conditions
Source: Front Microbiol. 2022 Nov 25;13:1044464. doi: 10.3389/fmicb.2022.1044464 (PMC9732472; doi:10.3389/fmicb.2022.1044464)
Supplement: Supplementary file 1 [file Presentation_1.pdf]

SUPPLEMENTAL ONLINE INFORMATION

For publication in conjunction with the following:

Morphological physiological and transcriptional responses of the freshwater diatom *Fragilaria crotonensis* to elevated pH conditions

Brittany N. Zepernick[1], David J. Niknejad[1], Gwendolyn F. Stark[1], Alexander R. Truchon[1], Robbie M. Martin[1], Karen L. Rossignol[2], Hans W. Paerl[2], Steven W. Wilhelm[1*]

[1]Department of Microbiology, University of Tennessee, Knoxville, TN, 37996, USA

[2]Institute of Marine Sciences, University of North Carolina at Chapel Hill, Morehead City, NC 28557

***Correspondence:**
Steven W. Wilhelm, wilhelm@utk.edu, Telephone: 1-865-974-0665, Fax: 1-865-974-4007

**Supplemental Table 1**: Statistical analysis of *F. crotonensis* morphological features (mean area, mean biovolume, mean length, mean width, mean roughness, and mean avg. green) collected at T_f of the morphology assay as a function of pH. Statistical analyses performed using unpaired two-tailed *t*-tests.		
**Treatment comparison**	**Summary**	**P value**
pH 7.7 vs. pH 9.2 Area (μm^2)	**	p=0.0074
pH 7.7 vs. pH 9.2 Biovolume (μm^3)	*	p=0.0310
pH 7.7 vs. pH 9.2 Length (µm)	**	p=0.0017
pH 7.7 vs. pH 9.2 Width (µm)	ns	p=0.0553
pH 7.7 vs. pH 9.2 Roughness	***	p=0.0002
pH 7.7 vs. pH 9.2 Avg. Green Content	****	p<0.0001

**Supplemental Table 2**: Statistical analysis of *F. crotonensis* filament concentrations and Chlorophyll *a* autofluorescence collected at T_f of the photopigment assay as a function of pH. Statistical analyses performed using unpaired two-tailed *t*-tests.		
**Treatment comparison**	**Summary**	**P value**
pH 7.7 vs. pH 9.2 filament conc. ($filaments \cdot mL^{-1}$)	ns	p=0.1745
pH 7.7 vs. pH 9.2 Chlorophyll *a* (fsu)	****	p<0.0001

**Supplemental Table 3**: Statistical analysis of *F. crotonensis* normalized photopigment concentrations (Chlorophyllide *a*, Chlorophyll *a*, Chlorophyll *c*1*c*2, Total Chlorophyll *a*, β-carotene, Violaxanthin, Diadinoxanthin, Fucoxanthin, Neoxanthin) and photopigment ratios (Total Carotenoids/Total Chl *a*, β-car/ Total Chl *a*, Viola/ Total Chl *a*, Diadino/Total Chl *a*, Total Chl *a*/Chl *c*1*c*2) collected at T_f of the photopigment assay as a function of pH. Statistical analyses performed using unpaired two-tailed *t*-tests.		
**Treatment comparison**	**Summary**	**P value**
pH 7.7 vs. pH 9.2 Chlorophyllide *a* ($fg \cdot \mu m\ fil^{-1}$)	ns	p=0.8657
pH 7.7 vs. pH 9.2 Chlorophyll *a* ($fg \cdot \mu m\ fil^{-1}$)	ns	p=0.3263
pH 7.7 vs. pH 9.2 Chlorophyll *c*1*c*2 ($fg \cdot \mu m\ fil^{-1}$)	ns	p=0.9484
pH 7.7 vs. pH 9.2 Total Chlorophyll *a* ($fg \cdot \mu m\ fil^{-1}$)	ns	p=0.3503
pH 7.7 vs. pH 9.2 β-carotene ($fg \cdot \mu m\ fil^{-1}$)	**	p=0.0015
pH 7.7 vs. pH 9.2 Violaxanthin ($fg \cdot \mu m\ fil^{-1}$)	**	p=0.0090
pH 7.7 vs. pH 9.2 Diadinoxanthin ($fg \cdot \mu m\ fil^{-1}$)	*	p=0.0189
pH 7.7 vs. pH 9.2 Fucoxanthin ($fg \cdot \mu m\ fil^{-1}$)	ns	p=0.4594
pH 7.7 vs. pH 9.2 Neoxanthin ($fg \cdot \mu m\ fil^{-1}$)	ns	p=0.3307
pH 7.7 vs. pH 9.2 Total Carotenoids/Total Chl *a*	ns	p=0.2207
pH 7.7 vs. pH 9.2 β-car/ Total Chl *a*	***	p=0.0002
pH 7.7 vs. pH 9.2 Viola/ Total Chl *a*	**	p=0.0093
pH 7.7 vs. pH 9.2 Diadino/Total Chl *a*	***	p=0.0008
pH 7.7 vs. pH 9.2 Total Chl *a*/Chl *c*1*c*2	ns	p=0.4335

**Supplemental Table 4**: Statistical analysis of *F. crotonensis* PhytoPAM analyses ($Fv \cdot Fm^{-1}$, $rETR_{max}$, I_k) collected at T_f of the photo physiology assay as a function of pH. Statistical analyses performed using unpaired two-tailed *t*-tests.		
**Treatment comparison**	**Summary**	**P value**
pH 7.7 vs. pH 9.2 $Fv \cdot Fm^{-1}$	ns	p=0.3089
pH 7.7 vs. pH 9.2 $rETR_{max}$ (µmol $e^-/m^2 \cdot s$)	****	p<0.0001
pH 7.7 vs. pH 9.2 I_k (µmol photons/ $m^2 \cdot s$)	****	p<0.0001

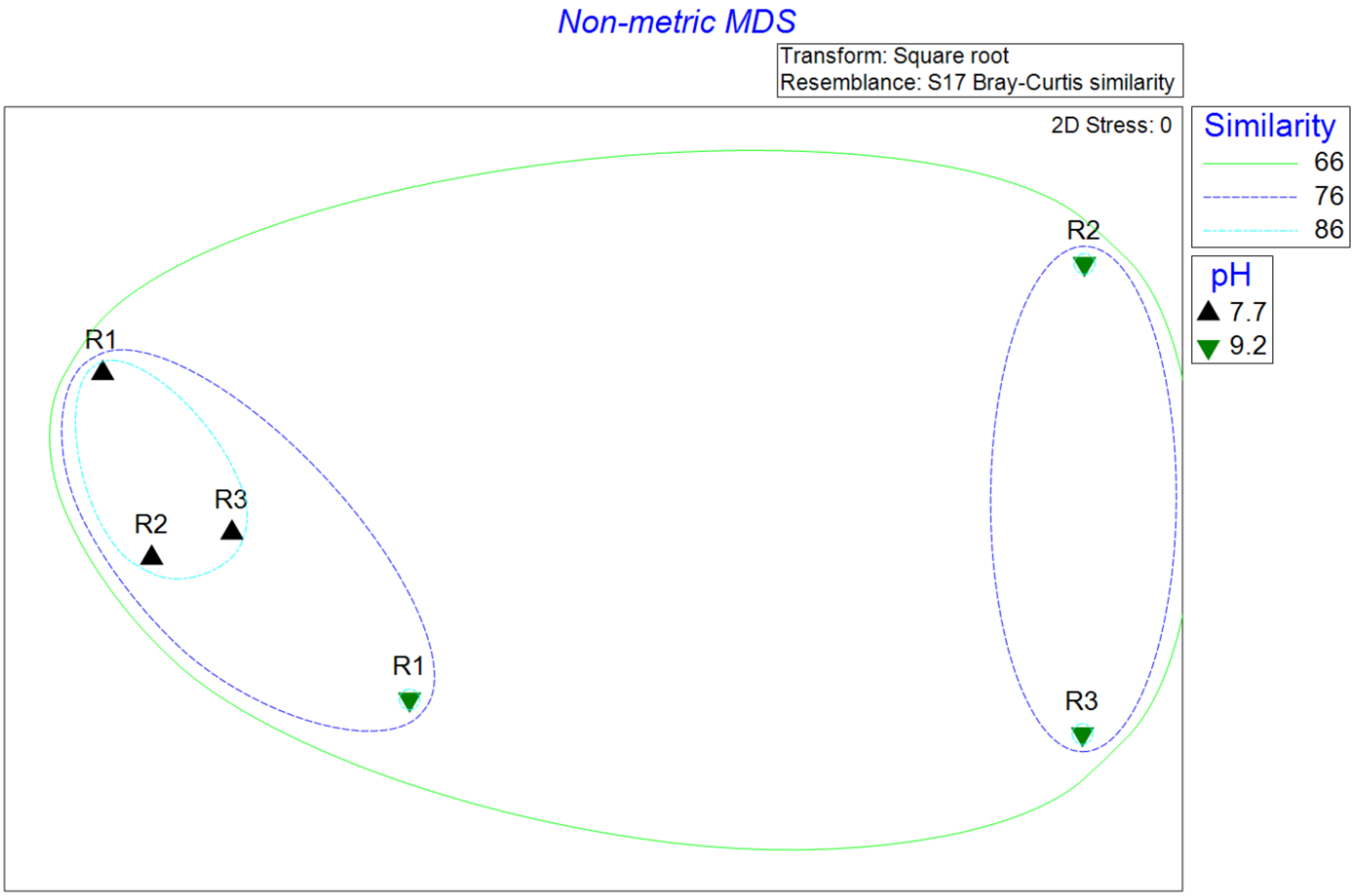

Supplemental Figure 1: Non-metric Multidimensional analysis (nMDS) of similarity between pH 7.7 and pH 9.2 transcriptomes (TPM). pH 7.7 replicates are indicated by black triangles, pH 9.2 replicates are indicated by inverted green triangles.

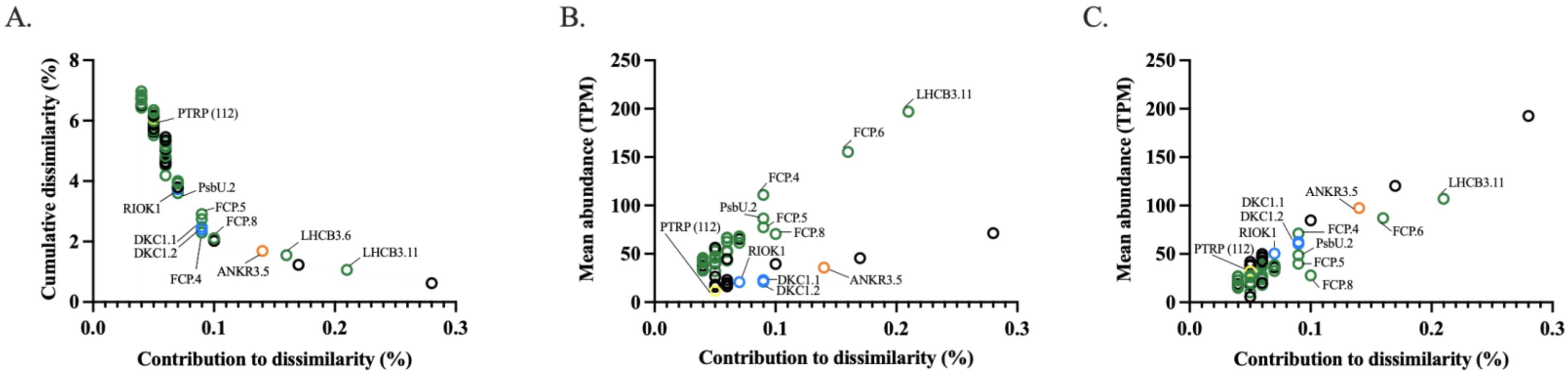

Supplemental Figure 2: Top 50 genes contributing to mean pH treatment dissimilarity (~27%) determined by Similarity Percentages (SIMPER). Genes categorized in COG category photosynthesis depicted in green, genes categorized in cell cycle control, cell division, chromosome partitioning depicted in blue, genes categorized in cell wall, membrane, envelope biogenesis depicted in orange, and genes categorized in mobilome: transposons; prophages depicted in yellow. Only annotated genes were included in final reports. (A) Contribution to dissimilarity (%) and cumulative dissimilarity (%) of the top 50 annotated genes driving mean dissimilarity between pH 7.7 and pH 9.2 normalized (TPM) expression values. (B) Mean abundance (TPM) of top 50 genes driving dissimilarity within the pH 7.7 replicates. (C) Mean abundance (TPM) of top 50 genes driving dissimilarity within pH 9.2 replicates.

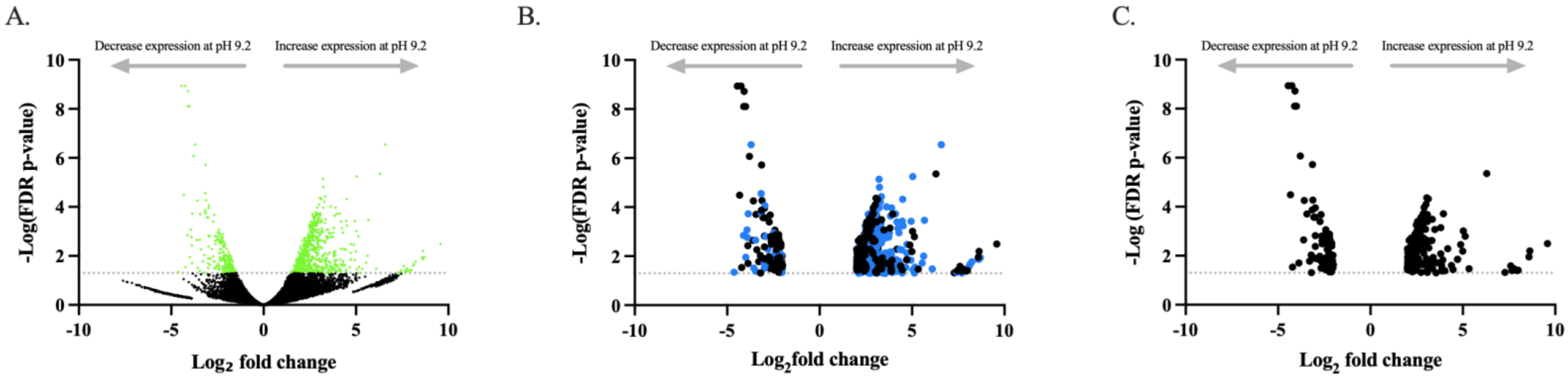

Supplemental Figure 3: Volcano plots of differentially expressed genes at pH 9.2 compared to pH 7.7. (A) All genes within the pH transcriptome. (B) All 713 DE genes according to the statistical cutoff (FDR-corrected p-value ≤ 0.05, $\log_2$ |fold-change| > 2). (C) All 435 DE and annotated genes with an assigned function used for all downstream analyses in this study.

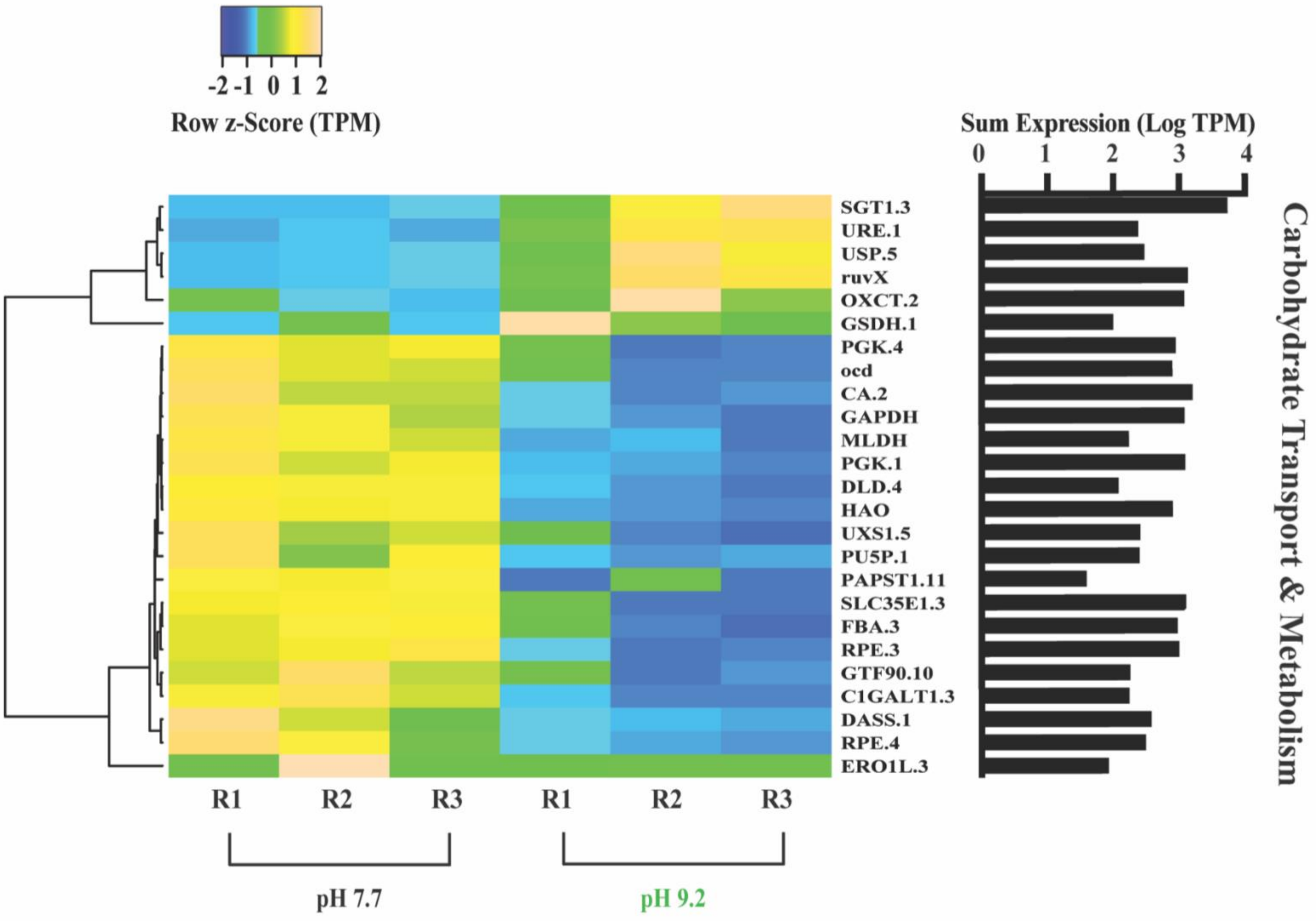

Supplemental Figure 4: Heat map depicting differentially expressed genes relating to the "Carbohydrate transport and metabolism" COG category. All TPM values were row z-scored, with increases in proportional transcript abundance indicated in yellow, and decreases in proportional transcript abundance indicated in blue. The sum of transcripts across all treatments (LogTPM) is indicated for each gene.

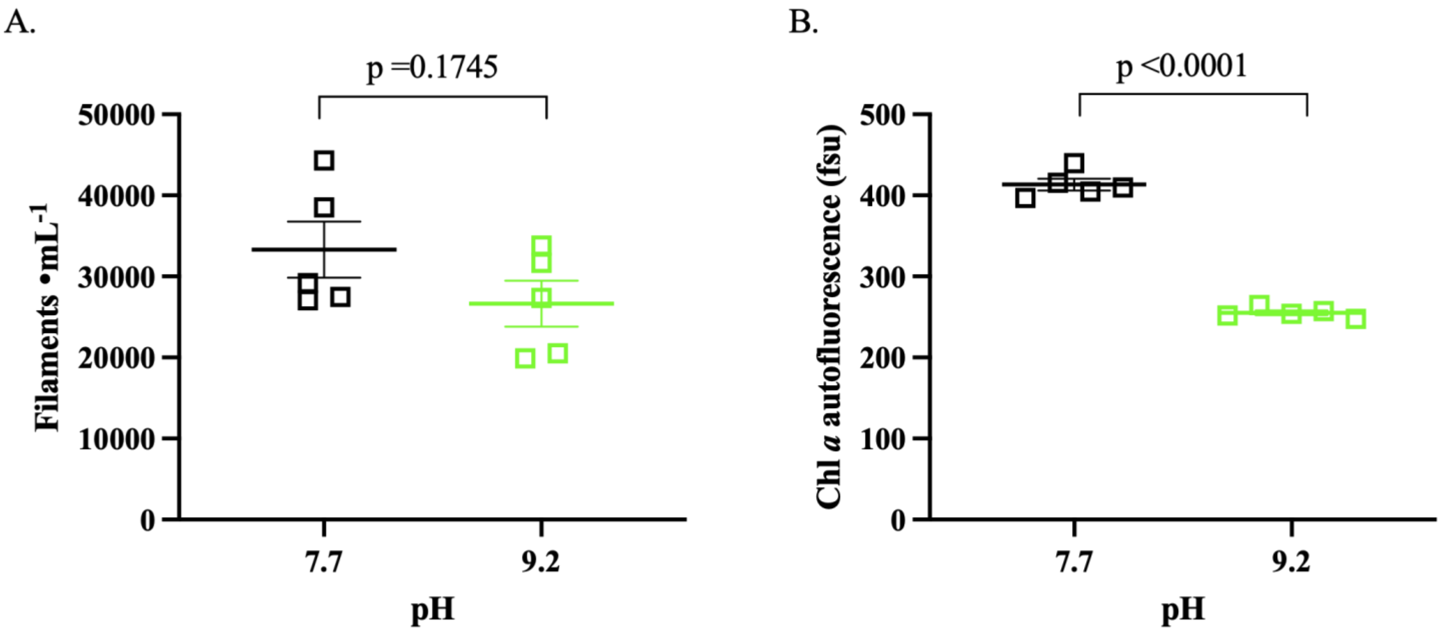

Supplemental Figure 5. Flow cytometry and fluorometer analyses corresponding to the photopigment pH assay results collected at (T_f). pH 7.7 replicates denoted by open, black squares. pH 9.2 replicates denoted by open, green squares. (A) *F. crotonensis* concentration (filaments • mL^{-1}). (B) *F. crotonensis* Chl *a* autofluorescence (fsu). Mean values indicated by solid horizontal lines and error bars represent the standard error of the mean (SEM).

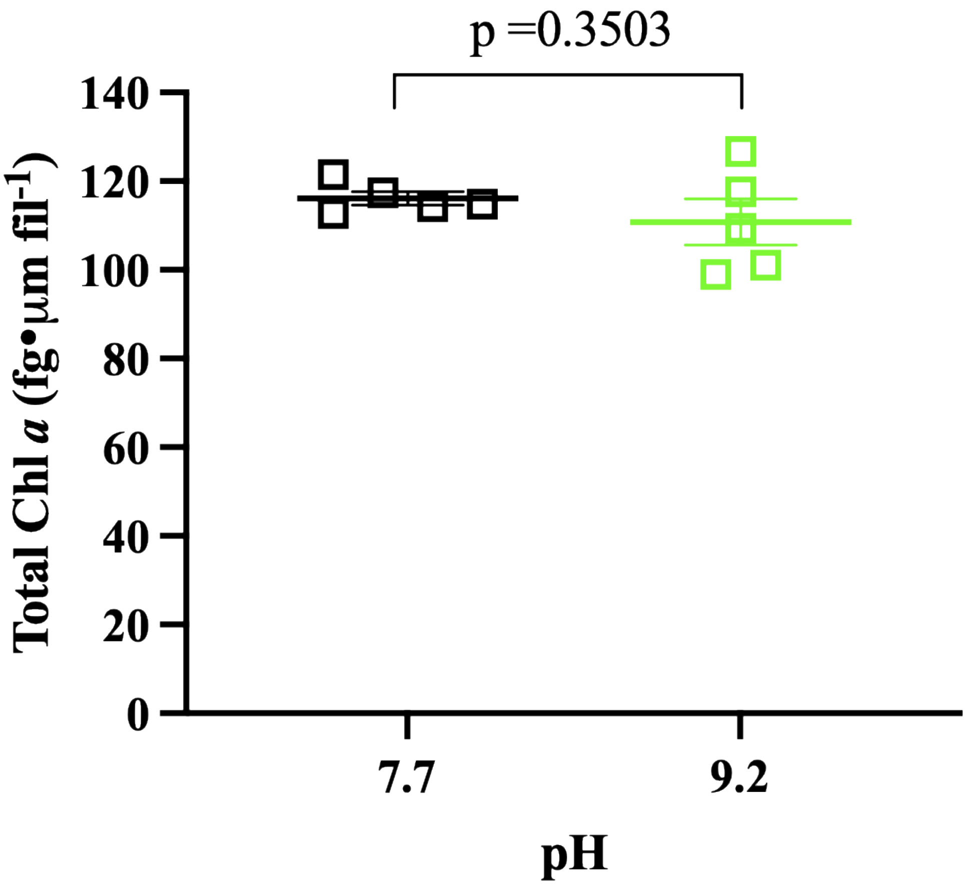

Supplemental Figure 6: Total Chlorophyll *a* photopigment pH assay results collected at (T_f). pH 7.7 replicates denoted by open, black squares. pH 9.2 replicates denoted by open, green squares. (A) Total Chlorophyll *a* pigment concentration (fg • μm fil^{-1}) of *F. crotonensis* filaments. Mean values indicated by solid horizontal lines and error bars represent the standard error of the mean (SEM).

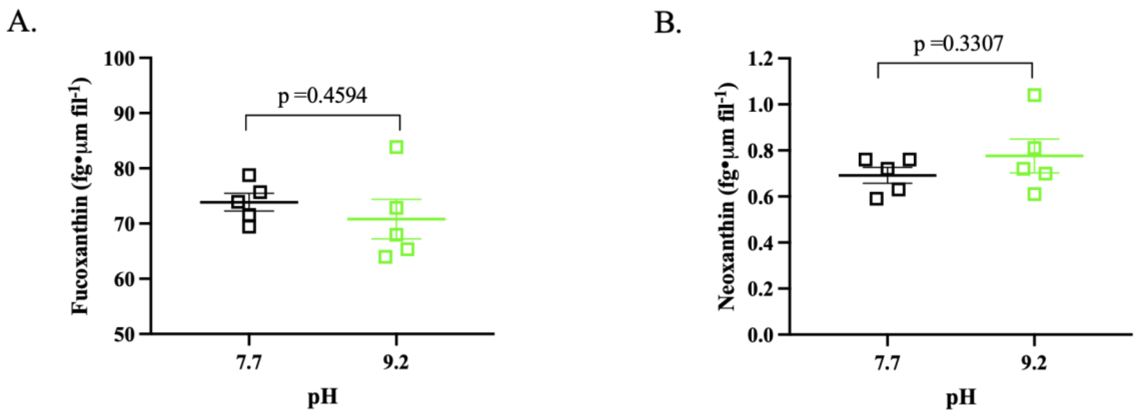

Supplemental Figure 7: Fucoxanthin and Neoxanthin photopigment pH assay results collected at (T_f). pH 7.7 replicates denoted by open, black squares. pH 9.2 replicates denoted by open, green squares. (A) Fucoxanthin pigment concentration (fg • µm fil^{-1}) of *F. crotonensis* filaments. (B) Neoxanthin pigment concentration (fg • µm fil^{-1}) of *F. crotonensis* filaments. Mean values indicated by solid horizontal lines and error bars represent the standard error of the mean (SEM).

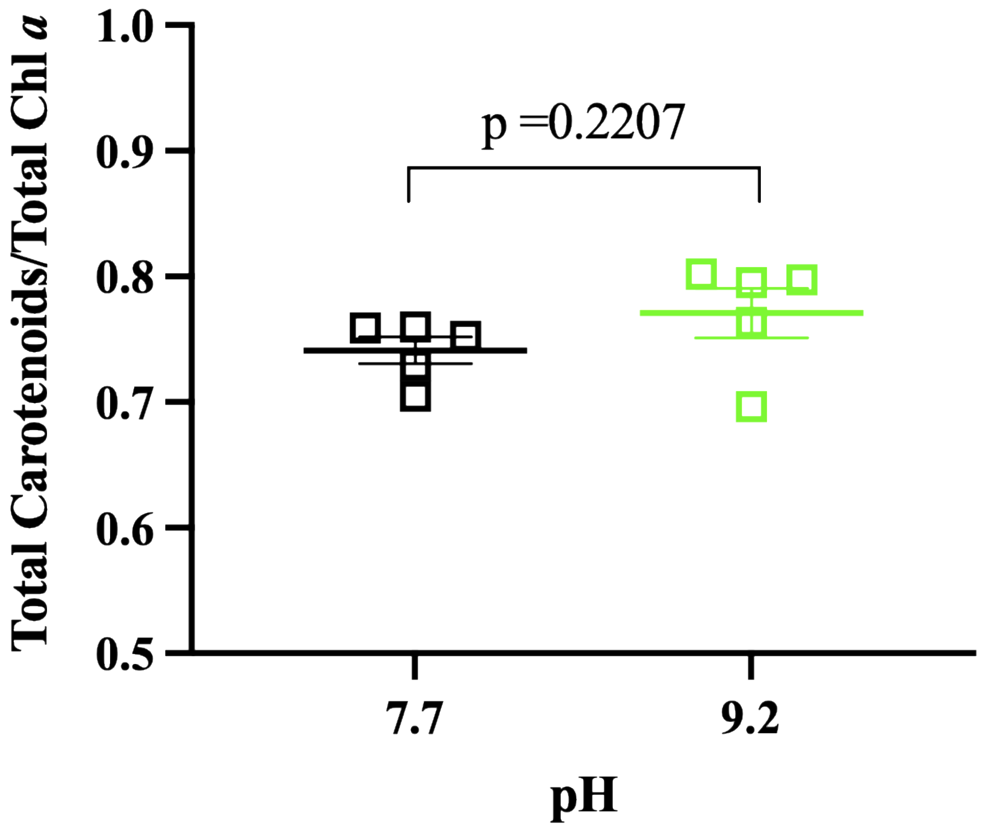

Supplemental Figure 8: Ratio of total carotenoid: total Chlorophyll *a* pigment in *F. crotonensis* filaments collected at (T_f). pH 7.7 replicates denoted by open, black squares. pH 9.2 replicates denoted by open, green squares. Mean values indicated by solid horizontal lines and error bars represent the standard error of the mean (SEM).

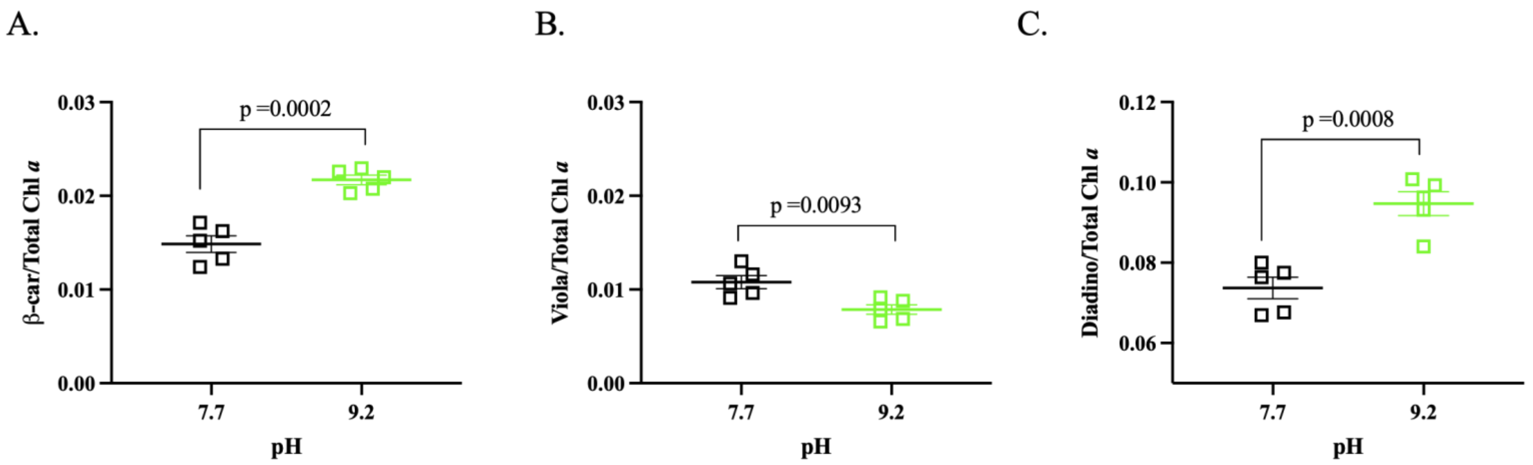

Supplemental Figure 9: Ratios of carotenoid pigments: total Chlorophyll *a* pigment in *F. crotonenis* filaments collected at (T_f). pH 7.7 replicates denoted by open, black squares. pH 9.2 replicates denoted by open, green squares. (A) Ratio of β-carotene: total Chlorophyll *a* pigment concentration in filaments. (B) Ratio of Violaxanthin: total Chlorophyll *a* pigment concentration in filaments. (C) Ratio of Diadinoxanthin: total Chlorophyll *a* pigment concentration in filaments. Mean values indicated by solid horizontal lines and error bars represent the standard error of the mean (SEM).

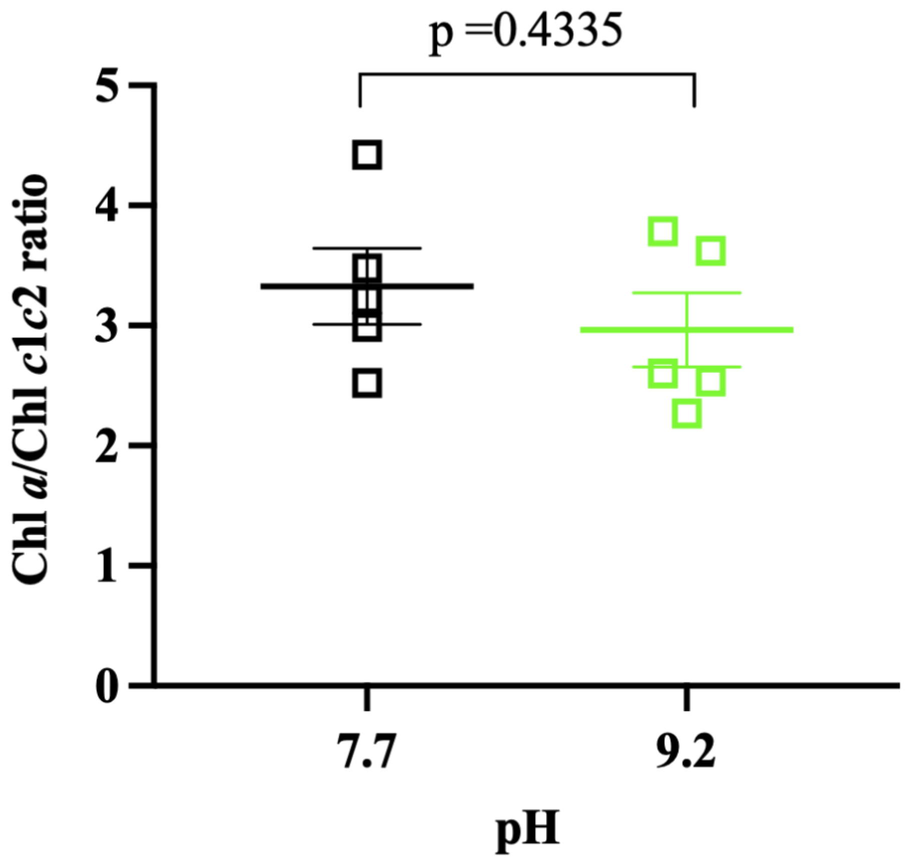

Supplemental Figure 10: Ratio of Chlorophyll *a:* Chlorophyll c^1c^2 pigment concentration in *F. crotonensis* filaments collected at (T_f). Mean values indicated by solid horizontal lines and error bars represent the standard error of the mean (SEM).
